# An Architecture for On-Line Measurement of the Tip Clearance and Time of Arrival of a Bladed Disk of an Aircraft Engine

**DOI:** 10.3390/s17102162

**Published:** 2017-09-21

**Authors:** José Miguel Gil-García, Alejandro Solís, Gerardo Aranguren, Joseba Zubia

**Affiliations:** 1 Department of Electronic Technology, University of the Basque Country, Nieves Cano 12, 01006 Vitoria-Gasteiz, Spain; 2 Department of Electronic Technology, University of the Basque Country, Alda. Urquijo s/n, 48013 Bilbao, Spain; alexsolismartinez93@gmail.com (A.S.); gerardo.aranguren@ehu.eus (G.A.); 3 Department of Communications Engineering, University of the Basque Country, Alda. Urquijo s/n, 48013 Bilbao, Spain; joseba.zubia@ehu.eus

**Keywords:** tip clearance, tip timing, real operation conditions measurement

## Abstract

Safety and performance of the turbo-engine in an aircraft is directly affected by the health of its blades. In recent years, several improvements to the sensors have taken place to monitor the blades in a non-intrusive way. The parameters that are usually measured are the distance between the blade tip and the casing, and the passing time at a given point. Simultaneously, several techniques have been developed that allow for the inference—from those parameters and under certain conditions—of the amplitude and frequency of the blade vibration. These measurements are carried out on engines set on a rig, before being installed in an airplane. In order to incorporate these methods during the regular operation of the engine, signal processing that allows for the monitoring of those parameters at all times should be developed. This article introduces an architecture, based on a trifurcated optic sensor and a hardware processor, that fulfills this need. The proposed architecture is scalable and allows several sensors to be simultaneously monitored at different points around a bladed disk. Furthermore, the results obtained by the electronic system will be compared with the results obtained by the validation of the optic sensor.

## 1. Introduction

Optimization and efficiency improvements in jet engines have the following advantages: a reduction in required fuel, a reduction in emissions released into the atmosphere, less demanding working levels of pressure and temperature, an extension of service life, and increments in the periodicity of the scheduled verification checks [1].

One of the methods employed to improve engine efficiency is to reduce the distance between the blade tip and the casing, the parameter called Tip Clearance (TC) [2]. The passing time at a known position of two consecutive blades is called the Time of Arrival (ToA) and is used to find out the vibration of a bladed disk. The state of the mechanics of a turbine engine can be obtained by measuring these two parameters [3].

The traditional way to measure the vibration has been to install strain gauges onto a small number of blades that are effectively monitored. This method has some drawbacks: the sensors interfere with the dynamics of the system, they measure the response of only those blades fitted with sensors, they break down easily, and the installation and setup time is too long [4]. Nowadays, sensors employed in these techniques are installed in the casing of the engine and provide contactless measurements. They have to withstand harsh conditions during the normal operation of the motor and be insensitive to the presence of substances produced by the combustion. Several sensors capable of non-intrusively measuring the ToA have been developed based on different physical principles: inductive [5], optic [6], capacitive [7], Eddy current [8], magneto-resistive [9], or microwave [10] sensors. Additionally, all these types of sensors are able to determine the TC for each blade, overcoming the limitations of the traditional electro-mechanical discharge sensor, which was able to measure only the distance to the closest blade [11].

Several authors [12,13,14] detail how the sensors’ output is converted into TC by applying calibration curves. These curves are calculated in the laboratory by measuring the sensor-conditioner output for different known distances from the sensor to the blade in the required range.

In order to obtain the ToA, a threshold level defines the instant at which the blade is in front of the sensor. This point can be detected in the positive edge of the waveform, when it reaches the maximum or when it falls back [15], as long as it is consistent over the whole process. Some sensors detect the change of blade by employing a voltage level comparator with a threshold [16], or when the detection of the blade passing signal finishes [17], whereas some others can configure the edge, thresholds, and trigger time [18].

From the measurement of the ToA, some alternative non-intrusive methods to calculate the state of vibration of a turbo engine have been developed. These methods are grouped together under the name of Blade Tip Timing (BTT) methods and allow for the deduction of the amplitude and frequency values of the vibration. Abdelrhaman et al. [19] recapitulate the methods used to monitor the state of a turbo engine. Diamond et al. [20] compare three BTT algorithms by using finite state models. Carrington et al. [21] run simulations based in the spring-mass-dump model trying to identify the vibration. Both his methods and the ones analyzed by Zielinski et al. [22] need to install several sensors around the bladed disk in order to characterize the vibrations.

In all referred cases, the parameters are obtained off-line, i.e., firstly the sensors output are registered and afterwards the waveforms are processed. Commercial instrumentation systems or dataloggers are the main election for storing the captured waveforms [23,24]. There are also some other descriptions based either on microcontrollers and communications systems [25,26] or on programmable logic devices (PLD) to compute the TC [27], to capture the ToA [28], or to detect flutter condition [29].

These parameters are measured in test rigs where the quality and performance of the manufactured turbine or compressor stages are tested [30]. Nevertheless, it is interesting to explore the possibility of measuring the TC and ToA of a motor under regular operating conditions, even when it is installed in an airplane. It has to be considered that the turbo-engines in flight suffer strong forces and accelerations that are the origin of transitory deformations of the blades. A system that implements these measures should be non-intrusive, autonomous, and able to measure the TC and ToA for each blade almost instantaneously in many points of the motor (on-line).

This article introduces an electronic architecture to measure the TC and ToA on-line based on an optic sensor. This architecture is scalable so that it can process various sensors simultaneously, compute the TC and ToA parameter, and prepare them for further post-processing.

Section 2 describes the architecture formed by the optic sensor and the processing electronics. Section 3 introduces a method to obtain the TC and ToA parameters. The test bench employed in the validation is introduced in Section 4. Section 5 shows the results obtained from the real signals. These results are analyzed in Section 6. Finally, Section 7 exposes the conclusion of this work.

## 2. Electronic System to Monitor a Bladed Disk

**Figure 1:**
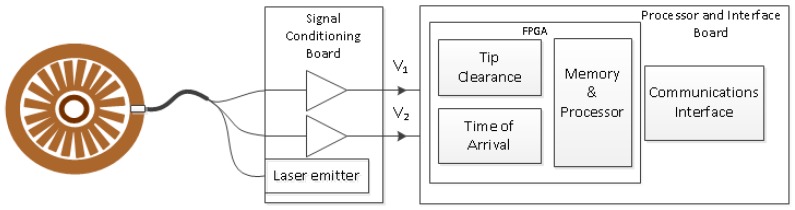
Architecture to monitor a bladed disk.

In order to measure the TC and ToA parameters, an electronic system depicted in Figure 1 has been developed. An optic sensor is installed in the casing of the engine with the bladed disk to monitor. A Signal Conditioning Board generates the laser that will illuminate the bladed disk. Another circuit in the Signal Conditioning Board receives the optic signals reflected by the blades and converts them into electrical signals. A system based on a commercial electronic board, the Processor & Interface Board, extracts the TC and ToA parameters from the digitized voltages and can transmit them to other processing devices. In the following paragraphs, each of these elements is described in detail.

**Figure 2:**
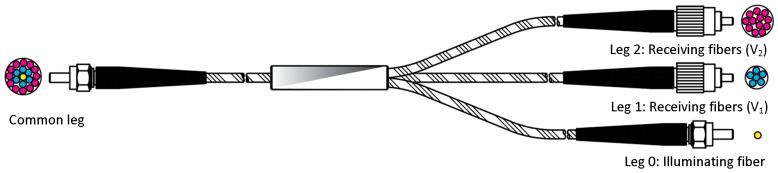
Trifurcated optic sensor.

The optic sensor has been designed ad hoc [31]. It consists in a trifurcated concentric optic fiber bundle. The central leg (yellow) has one single fiber and drives the laser light to the bladed disk. Part of the light gets reflected into the outer legs (blue and magenta), which drive the light to the signal conditioner. Figure 2 represents the described sensor.

**Figure 3:**
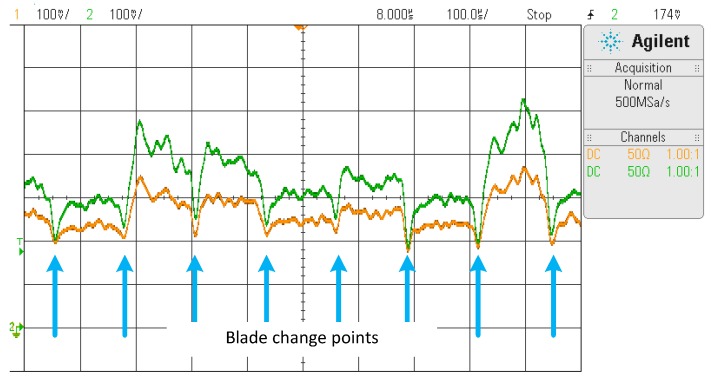
V_1_ (orange) and V_2_ (green) signals captured for seven blades.

Two transimpedance amplifiers in the Signal Conditioning Board convert the optic signal into voltages V_1_ (from the intermediate ring of fibers) and V_2_ (from the outermost ring of fibers). Figure 3 shows the waveforms V_1_ and V_2_ captured for seven blades. Blue arrows have been drawn where a blade change in front of the sensor is considered to facilitate the comprehension of the figure.

V_1_ and V_2_ signals are proportional to the optic signals representing the amount of light reflected by the blade captured by the sensor.

**Figure 4:**
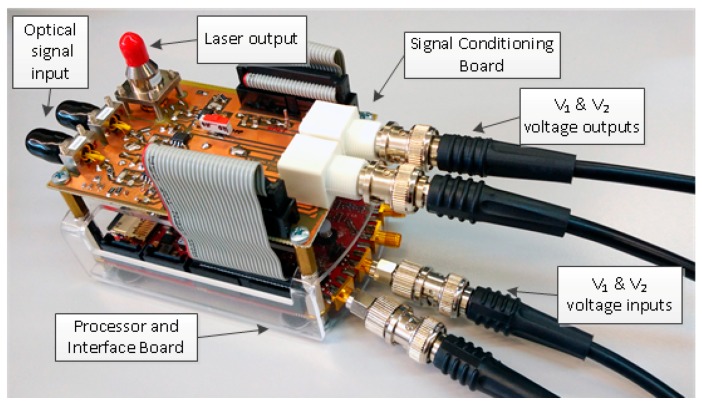
Electronic system to measure Tip Clearance (TC) and Time of Arrival (ToA).

TC and ToA parameter extraction from signals V_1_ and V_2_ has been implemented in a low-cost commercial development board called Red Pitaya [32], which will play the Processor and Interface Board role. This board has a Field Programmable Gate Array (FPGA) type of circuit with the capacity to integrate custom logic and a processor. V_1_ and V_2_ voltages are digitized by two high speed acquisition channels present in the Processor and Interface Board. They are two 14-bit bipolar channels in the range ±1 V and sampled at 125 MHz. An Ethernet interface for configuration, control and results inspection is also available. The whole system is displayed in Figure 4.

Two types of approaches can be carried out in order to determine TC and ToA: (1) capture in memory a time window of V_1_ and V_2_ waveforms and later calculate the value of the parameters (off-line) or (2) extract their values while the signals are read (on-line).

The off-line method is suitable for sequential processing that takes into consideration global values of the samples. Results are obtained after elapsing the acquisition and the processing time of the time window in memory.

The research in this article chooses the second approach. The on-line method employs pipeline processing techniques and is more suitable for processing several sensors simultaneously in parallel. TC and ToA are calculated immediately after the detection of a change of blade at the sensor position. The processor has been created as an intellectual property (IP) core written in a hardware description language. The IP core can be instantiated as many times as sensors are required to be processed. Once calculated, the parameters TC and ToA for the blade that just passed are available in memory for possible post-processing using BTT techniques, alarms generation, etc.

**Figure 5:**
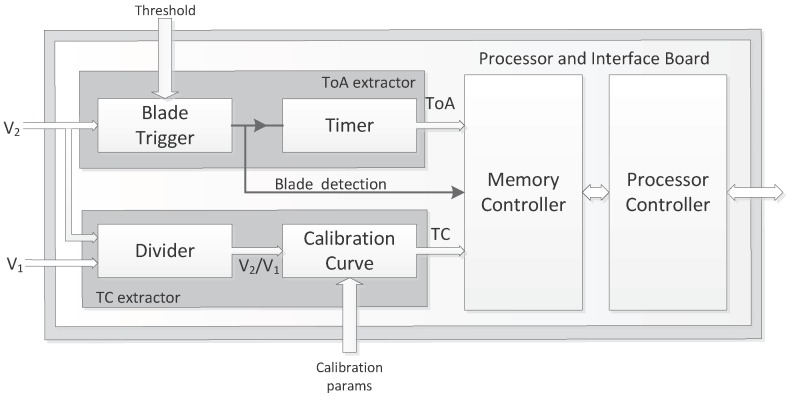
Architecture to measure TC and ToA with an optic sensor.

Figure 5 shows a block diagram of the TC and ToA processor implemented in the FPGA. It consists of three main blocks:

- a TC extractor;
- a ToA extractor;
- a Memory Controller and Processor Controller.

## 3. TC and ToA Parameter Extraction

TC extraction is accomplished in two steps: (1) divide the two input signals and (2) convert the ratio into distance. The reflected signals captured by the outer rings of the sensor depend on several factors, such as variations in the emitted light intensity, changes in the material reflectivity of the blades and optical loses. The quotient between V_2_ and V_1_ cancels out that dependency. Hence, in a certain range, the ratio is proportional to the distance between the sensor tip and the reflecting surface.

**Figure 6:**
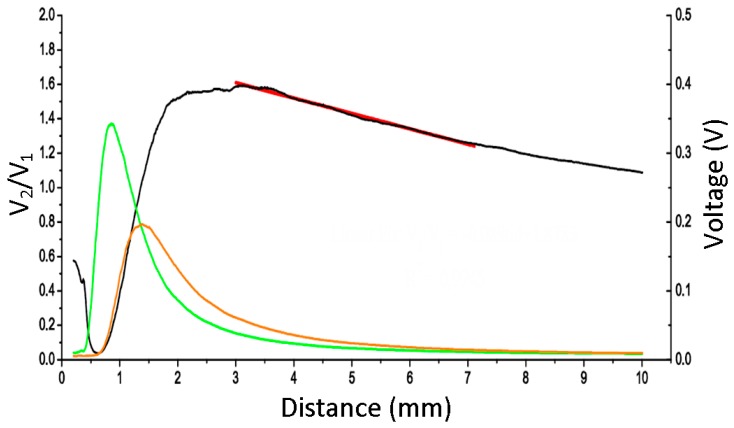
Calibration curve for the optic sensor.

Prior to making these measurements, it was required that a laboratory test was carried out on the sensor to obtain a relationship between the voltages and the TC. Figure 6 shows signals V_1_ (green), V_2_ (orange), and their ratio (black). As a result of the test, a straight line (red) was used as a calibration fit.

V_2_/V_1_ = A·d + B. (1)

The obtained value of the slope A was −0.08969 and the intercept B was 1.8783 for the tested sensor. Letter d stands for distance. The linear fit was obtained by the least-square method with a coefficient of determination R^2^ = 0.9945. It is valid in the 3–7 mm range.

The 16-bit by 16-bit division has been implemented in the pipeline mode. The ratio is introduced in the linear fit obtained in the calibration performed in the laboratory in order to obtain the TC (calibration curve block). The slope and intercept of the linear fit in Equation (1) are transformed so that they can be operated digitally, which yields TC in Equation (2).


TC = 351703625 − 22857 × V_2_/V_1_. (2)

The divider has 58 clock pulses latency for the first calculated ratio, but one new ratio is calculated for each additional clock pulse afterwards. Without the loss of generality, the 16-bit values corresponding to the TC are stored in memory in fixed point representation.

The slope and intercept values of the linear fit can be configured before starting the measure to adapt the calculation to different sensors or situations.

ToA determination requires that only one signal is analyzed over the time period. V_2_ is chosen because of its larger dynamic range. The algorithm finds the instant at which there is a change of blade in front of the sensor corresponding with the minimum of V_2_. Relative minima found should be disregarded due to the lack of monotonicity because of the defects in the blades or sensing defects.

**Figure 7:**
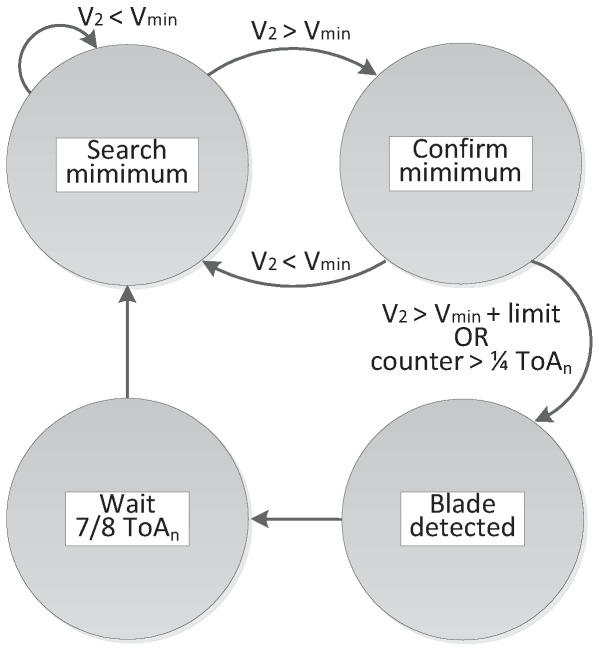
Pseudo-state machine to detect global minima.

In order to confirm that a minimum is due to a change of blade, the algorithm in Figure 7 has been followed. Firstly, the increase of V_2_ is awaited. From that instant, the search of a minimum is started (search minimum state). If V_2_ goes over a threshold level or no smaller minimum is found over one fourth of the last measured ToA period, the considered minimum is regarded as a change of blade (confirm minimum state). Once detected, the detection signal is activated (blade detected state). Then, the temporal value at which the minimum is confirmed is stored and considered to be the present ToA. No new minima are searched until seven-eighths of the measured ToA have elapsed (wait 7/8 ToA_n_ state), as ToA is not considered able to change sharply.

During the first turns of the bladed disk under study, the algorithm acts in learning mode where it calculates the average ToA, ToA_n_, which will be used in the next detection. Parallel to the search of the next ToA, the average ToA of the last blade is calculated as the weighted average of the currently stored value, ToA_n−1_, and the last detected ToA. As expressed in Equation (3), a weight of seven to the stored value and a weight of one to the last detected value have been proven to be adequate. In this way, the algorithm adapts itself to changes in the speed of the bladed disk.

(3) $${\mathrm{ToA}}_{n}=\frac{7\times {\mathrm{ToA}}_{n-1}+\mathrm{ToA}}{8}.$$

**Figure 8:**
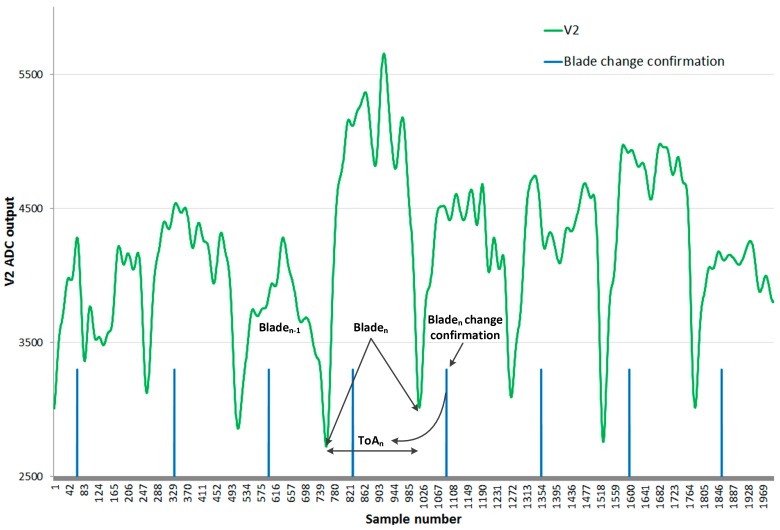
Change of blade confirmation.

All of these conditions comprise the algorithm for finding the values of ToA without any previous knowledge of the nominal rotational speed that the engine is under. Figure 8 shows the V_2_ ADC output (green) corresponding to a real test. The vertical lines (blue) correspond to the instant where the last measured minimum is confirmed to be a change of blade. The ToA can then be calculated, as the V_2_ waveform local minima are continuously monitored. The change of blade confirmation is not under the minimum because of the delay imposed by pipeline processing, but it can be noted that it happens before the current blade finishes passing.

TC and ToA parameters are calculated for each blade of the bladed disk. At any time, all values can be reset if a sudden change in the rotational speed renders the stored values obsolete. The processor is able to store the information of a bladed disk with up to 2048 blades. Before starting to monitor the test, it is required that the IP core is configured with the number blades in the bladed disk.

## 4. Tests

**Figure 9:**
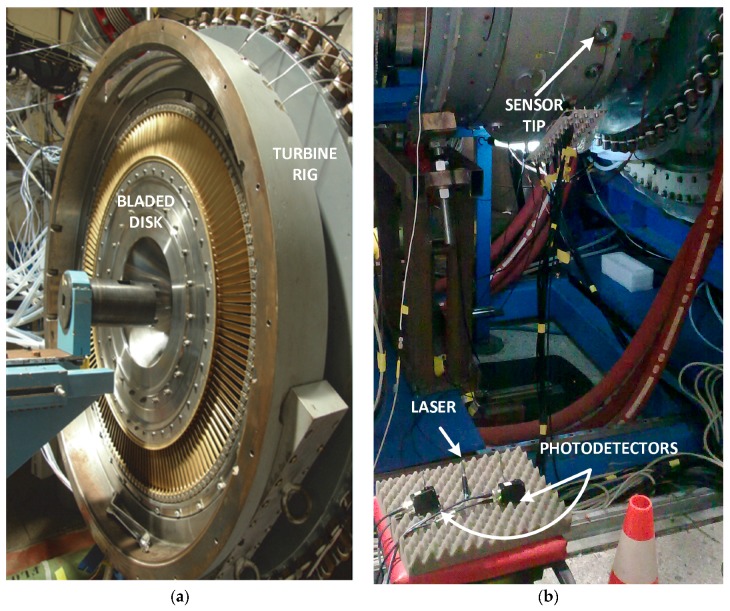
(**a**) Compressor disk with 146 blades in the turbine rig; (**b**) sensor test at the Aeronautical Technology Center (CTA).

The sensor was validated at the facilities of the Aeronautical Technology Center (CTA, located in Miñano, Álava, Spain), a research center specialized in aeronautical testing on structures and systems. The sensor has been tested in a wind tunnel measuring the 146 blades compressor stage of 528.3 mm, depicted in Figure 9a. The CTA procedures for that disk were followed in the test reaching up to 5000 revolutions per minute (rpm). Its readings have been compared with those from the discharge sensor usually employed at CTA. Figure 9b corresponds to one of those tests where commercial equipment was used for the laser and the photodetectors. 

V_1_ and V_2_ waveforms sampled at 2 MHz were stored in an oscilloscope under different rotational speeds. Those signals were post-processed off-line in the laboratory with a computer to obtain the TC and ToA following the next steps:

- Ten consecutive spins of samples of V_2_ and V_1_ were selected.
- They were filtered with a cutoff frequency of 50 kHz.
- The change of blade instant was determined by detecting when the second derivative of V_2_ overcame a certain threshold.
- For each spin, the minimum TC was calculated.
- The average minimum TC for the 10 spins was also calculated.

When measuring TC, the sensor was shown to differ from the commercial discharge sensor employed in the company by less than 2.22% in the worst case. To validate the proper functioning of the electronic system and the proposed architecture, the same set of signals employed to validate the optic sensor was applied. They correspond to stationary rotational speeds of 3225, 4373, and 4608 rpm. For each speed, the following set of signals has been generated:

1. one single turn with the original signals sampled at 2 MHz (one-turn-original set);
2. one single turn with filtered signals with a cutoff frequency of 50 kHz (one-turn-filtered set);
3. ten consecutive turns with filtered signals with a cutoff frequency of 50 kHz (ten-turn-filtered set).

The first set of digitized signals (one-turn-original) was also employed in simulations run in a computer to validate the ability of the algorithms to correctly detect the change of blade instants and the minimum TC for each blade. In this way, the theoretical values that the electronic systems should obtain were computed, and the impact of digitizing the calibration curve and the V_2_ and V_1_ waveforms with respect to the off-line processing was determined.

**Figure 10:**
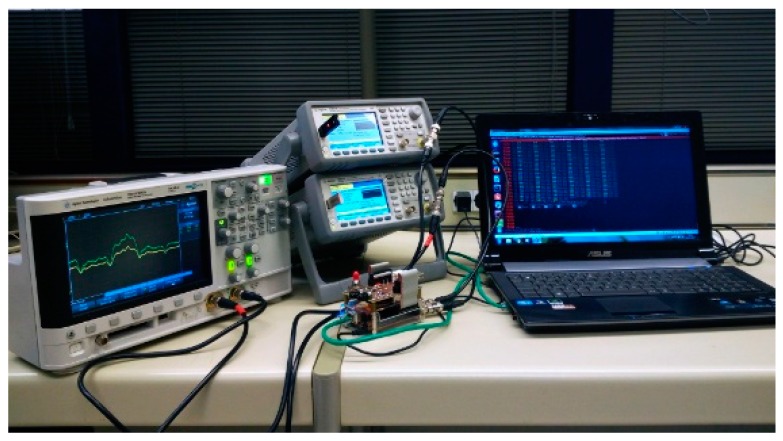
Validation test bench.

Once the algorithm was validated by simulation, the first two sets of signals (one-turn-original and one-turn-filtered) were played in two synchronized arbitrary function generators from Agilent model 33521A to emulate the measurement of real signals. The function generators outputs were connected to the Processor and Interface Board inputs to emulate that the same turn was measured over and over by the electronic system. Finally, with the third set of signals (ten-turn-filtered), the TC and ToA processor was reconfigured to store 10 consecutive turns of 146 blades. While the system is running, the measured parameter values can be displayed in a computer on-line. The test bench is shown in Figure 10.

## 5. Results

**Figure 11:**
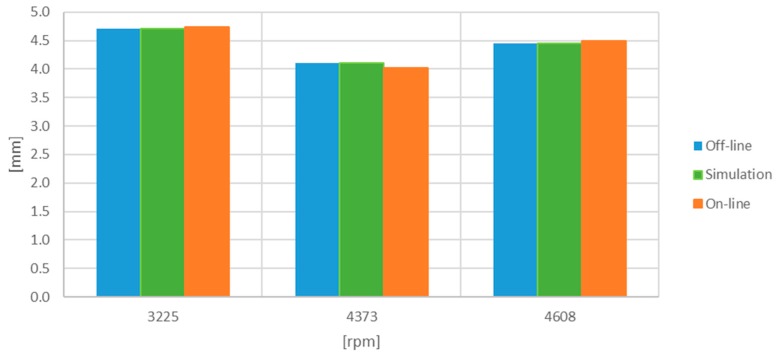
Minimum TC [mm] obtained with the one-turn-original set of signals.

**Figure 12:**
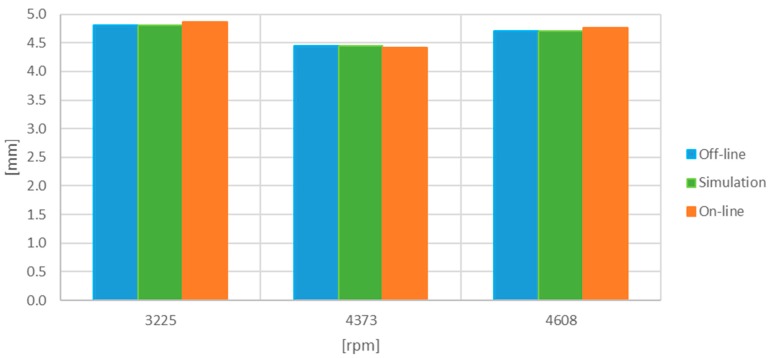
Minimum TC [mm] obtained with the one-turn-filtered set of signals.

The obtained results are tabulated in Table 1 and Table 2 and plotted in Figure 11 and Figure 12. They show the results corresponding to the off-line processing of a computer, the simulations with digitized signals, and the values obtained in the on-line emulation. The deviation percentage from the off-line processing is also shown. The results from the emulation were corrected by 6% due to the uncertainty in the analogue sub-system of the Red Pitaya (DC offset error less than 5% and maximum gain error less than 10% full scale) and because the linear fit was obtained for an Agilent 34410A multimeter rather than for the Processor and Interface Board. 

**Figure 13:**
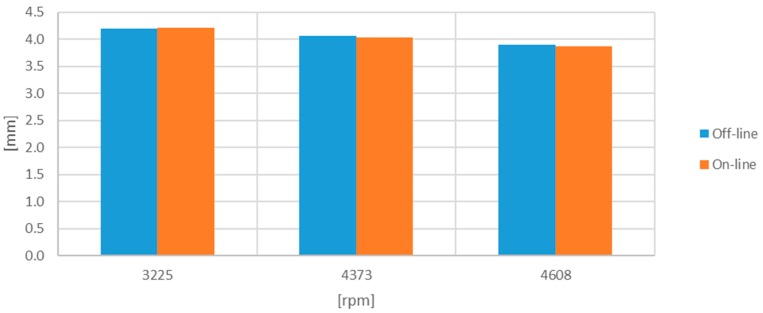
Minimum TC [mm] obtained with the ten-turn-filtered set of signals.

**Figure 14:**
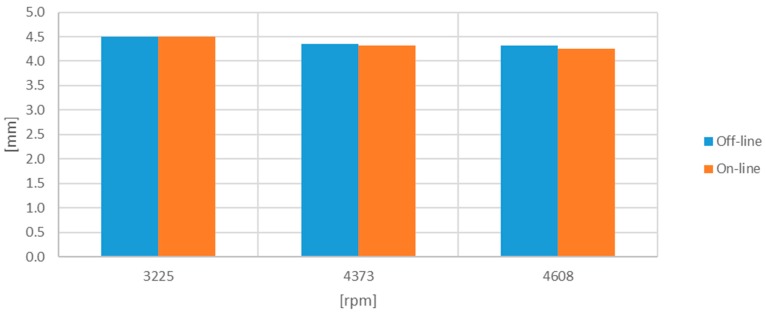
Average minimum TC [mm] obtained with the ten-turn-filtered set of signals.

With the third set of signals, the TC and ToA processor was reconfigured to store 1460 blades corresponding to the 10 consecutive turns. The results are shown in Table 3 and Table 4 and plotted in Figure 13 and Figure 14.

Table 3 shows the minimum TC obtained over the 10 turns, whereas Table 4 shows the average of the minimum TC obtained in each of the 10 turns. In this case, the results are obtained from the filtered signals because the original study was based on these signals only.

The ToA parameter changes from turn to turn due to the vibration. However, the sum of all ToAs shows whether all blades are captured and what the spin speed is. Table 5 shows the measured rotational speeds.

## 6. Analysis of Results

Off-line and simulation values for TC are very close. The difference is due to the digitalization of the samples and linear fit. The maximum error due to the digitalization of the calibration curve represents 0.002 mm with respect to off-line processing with analogue values. This is acceptable if we take into account that sensor accuracy is considered to be 0.024 mm.

**Figure 15:**
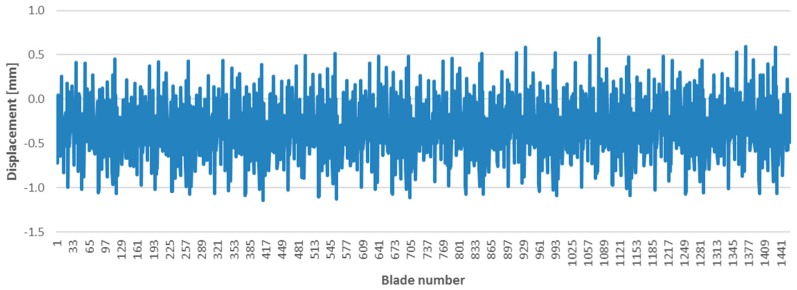
Displacement for 1460 blades (10 consecutive turns).

On-line processing was able to detect all blade changes and the sum of all ToAs is coherent with the off-line processing. Figure 15 was produced to show the displacements measured for 1460 blades (10 turns) rotating at 3225 rpm.

**Figure 16:**
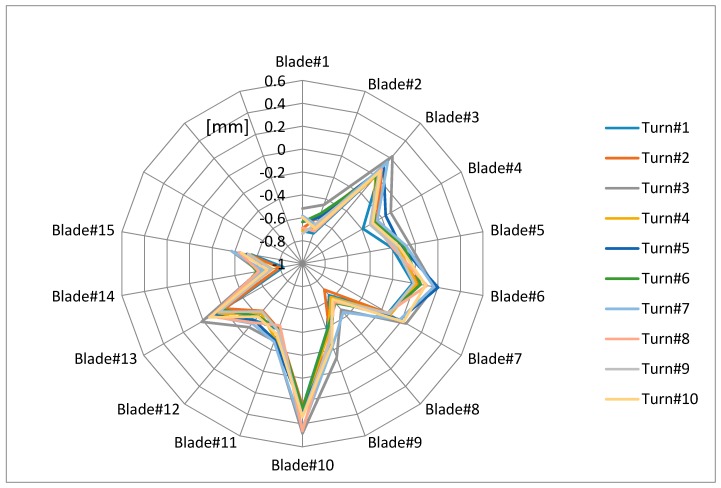
Deflections for 15 blades and 10 consecutive revolutions.

Figure 16 shows the calculated deflections for 10 turns of the 15 first blades. Unless the vibration frequencies of the blades are multiples of the rotation frequency, the measured deflections change in consecutive turns for the same blade. It can be seen how deflections are in the same range and are coherent from turn to turn.

The system is able to correctly detect the change of blade, as shown in Figure 8. The blue line indicates the instant that triggers the store of the values in memory. Hence, it is able to process the last blade before the current blade finishes passing in front of the sensor.

The system is capable of measuring continuously the TC and ToA for each blade of a bladed disk. It is also able to store statistical data of those parameters that can be used to activate alarms or foresee behavioral trends of the monitored stage.

Regarding the electronic system features, the digitalization process only has a 2 µm contribution to the uncertainty of the sensor accuracy, deemed to be 24 µm, in the determination of TC. ToA resolution is 8 ns due to the 125 MHz sampling rate of the converters. This frequency determines the minimum deflection that can be detected, which is also function of the radius of the bladed disk, e.g., in the case of the bladed disk rotating at 3226 rpm, the displacement resolution would be 1.4 µm, much smaller than the resolution achieved by the 2 MHz sampling rate used in the off-line process.

The proposed architecture is scalable as long as there are enough analogue channels in the system. In the implementation (Zynq 7010), the most used resource was the memory interface (BRAM), as it reserved memory for 2048 blades. However, this can be tuned for a concrete turbine, or the circuit can be redesigned in order to be installed onboard an aircraft.

## 7. Conclusions

An architecture in which the TC and ToA parameters of a turbine can be measured in real time is proposed. This architecture was implemented for the measurement of those parameters with a trifurcated optic sensor by an electronic circuit based on an FPGA.

The proposed architecture can be instantiated many times to process several sensors simultaneously. It makes the parameter values immediately available for a post-processor or alarm activation. 

A new method used to determine the ToA with this sensor is also proposed. It adapts itself automatically to the rotation speed. This method, together with the pipeline implementation of the TC calculation, is more suitable for the FPGA circuit than the one used in off-line processing. The values obtained for the TC and ToA in the tests are comparable, once corrected, with results yielded by the off-line processing. 

TC monitoring can drive design decisions in order to improve engine efficiency while maintaining safety. It allows, together with the ToA parameter, for the functional assessment of a manufactured bladed disk. 

The blade deflection calculated on-line from the ToA parameter allows for the monitoring of the structural health of the blades and can detect malfunction conditions of the engine.

After conducting all the experiments, the hardware platform used—or an equivalent one—has been shown to be robust and flexible. Similar hardware, provided with enough analogue channels, will allow for the measurement of the TC and ToA parameters of a bladed disk with multiple sensors autonomously and in real time.

## Figures and Tables

**Table 1:** Minimum TC [mm] obtained with the one-turn-original set of signals.

Working Point [rpm]	Off-Line [mm]	Simulation (% Dev.) [mm]	On-Line (% Dev.) [mm]
3225	4.706	4.704 (−0.04)	4.741 (0.74)
4373	4.099	4.099 (0.00)	4.023 (−1.8)
4608	4.456	4.456 (0.01)	4.486 (0.67)

**Table 2:** Minimum TC [mm] obtained with the one-turn-filtered set of signals.

Working Point [rpm]	Off-Line [mm]	Simulation (% Dev.) [mm]	On-Line (% Dev.) [mm]
3225	4.813	4.809 (−0.07)	4.865 (1.09)
4373	4.451	4.451 (0.01)	4.410 (−0.90)
4608	4.703	4.702 (−0.02)	4.760 (1.21)

**Table 3:** Minimum TC [mm] obtained with the ten-turn-filtered set of signals.

Working Point [rpm]	Off-Line [mm]	On-Line (% Dev.) [mm]
3225	4.198	4.213 (0.35)
4373	4.070	4.035 (−0.86)
4608	3.894	3.876 (0.46)

**Table 4:** Average minimum TC [mm] obtained with the ten-turn-filtered set of signals.

Working Point [rpm]	Off-Line [mm]	On-Line (% Dev.) [mm]
3225	4.505	4.496 (0.21)
4373	4.352	4.320 (0.73)
4608	4.318	4.260 (1.33)

**Table 5:** Average rotation speed obtained for 10 revolutions with filtered signals.

Working Point [rpm]	Off-Line [mm]	Std. Dev. [rpm]	On-Line [rpm]	Std. Dev. [rpm]	% Dev.
3225	3226.47	0.28	3226.78	0.21	0.01
4373	4372.46	1.09	4372.48	0.40	0.00
4608	4609.21	0.62	4609.69	0.70	0.01

## Abbreviations

The following abbreviations are used in this manuscript:

BRAM	Block Random Access Memory
BTT	Blade Tip Timing
CTA	Aeronautical Technology Center (According to its initials in Spanish)
DC	Direct Current
FPGA	Field Programmable Gate Array
IP	Intellectual Property
PLD	Programmable Logic Device
rpm	revolutions per minute
TC	Tip Clearance
ToA	Time of Arrival
